# Lobaplatin-Induced Apoptosis Requires p53-Mediated p38MAPK Activation Through ROS Generation in Non-Small-Cell Lung Cancer

**DOI:** 10.3389/fonc.2019.00538

**Published:** 2019-07-24

**Authors:** Hongming Zhang, Runzhe Chen, Xiyong Wang, Haijun Zhang, Xiaoli Zhu, Jibei Chen

**Affiliations:** ^1^Department of Respiratory Medicine, The Affiliated Yancheng Hospital, Medical School, Southeast University, Yancheng, China; ^2^Department of Hematology and Oncology, Zhongda Hospital, Medical School, Southeast University, Nanjing, China; ^3^Anhui Medical University (Suzhou Municipal Hospital), Suzhou, China; ^4^Department of Pulmonary Medicine, Zhongda Hospital, Medical School, Southeast University, Nanjing, China

**Keywords:** lobaplatin, non-small-cell lung cancer, p53, reactive oxygen species, p38MAPK, apoptosis

## Abstract

Platinum-based chemotherapy is recommended as the first-line treatment regimen for patients with advanced non-small-cell lung cancer (NSCLC). Lobaplatin (LBP), a third-generation platinum anti-neoplastic agent, has shown an improved efficacy. This study is aimed to investigate the mechanisms of LBP-induced apoptosis in the A549 p53 wild-type cell line. The Cell Counting Kit-8 assay (CCK-8), flow cytometry (FCM), Western blot, xenograft tumor models, terminal deoxynucleotide transferase dUTP nick end labeling (TUNEL), and RNA interference were used in this study. Our results showed that the proliferation of A549 cells could be inhibited by LBP. At lower concentrations, LBP triggered cell cycle arrest at the G1 phase in A549 cells. LBP could also induce apoptosis of A549 cells. LBP also increased the expression of PARP and Bax and the cleavage of caspase-3, caspase-8, and caspase-9 and reduced Bcl-2 expression. *In vivo* experiment confirmed that LBP could inhibit tumor growth in the A549 xenograft models and induce apoptosis. Apoptosis of A549 cells was decreased after transfected with p53 shRNA or treated with reactive oxygen species inhibitor NAC and p38MAPK inhibitor SB203580, suggesting that the p53/ROS/p38MAPK pathway appeared to mediate the LBP-induced apoptosis of A549 cells. Our data demonstrate that LBP could be a promising candidate for the treatment of NSCLC with wild-type p53.

## Introduction

Lung cancer is the leading cause of cancer-related death worldwide (1), with non-small-cell lung cancer (NSCLC) accounting for 85% of lung cancer cases (2). Platinum-based doublet regimens have been used as a standard first-line therapy against NSCLC. There are two kinds of platinum-based agents widely used in NSCLC: cisplatin (DDP) and carboplatin (CBP). However, severe toxicity and drug resistance after utilization often occur. Therefore, exploration of new effective platinum-based agents with low toxicity for the treatment of NSCLC is urgently required.

**Figure 1:**
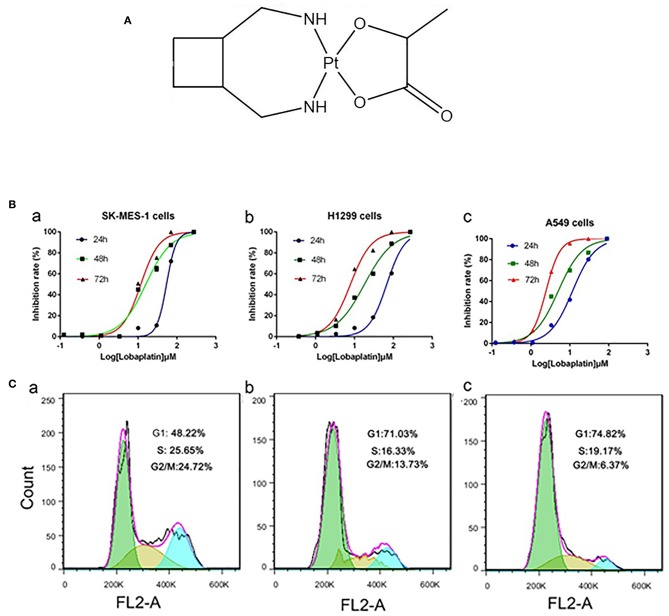
LBP inhibited the proliferation of human NSCLC cells and induced the cell cycle progression of A-549 cells. **(A)** Chemical structure of LBP. **(B)** Cells were exposed to various concentrations of LBP for 24, 48, or 72 h followed by analysis with a CCK-8 assay. **(a)** SK-MES-1, **(b)** NCI-H1299, **(c)** A-549. All of the assays were performed in triplicate. All of the assays were performed in triplicate. **(C)** A549 cells were exposed to various concentrations of LBP **(a)** 0 μM, **(b)** 6 μM, and **(c)** 12 μM for 24 h followed by analysis of the cell cycle by FCM.

Lobaplatin (LBP) is a third-generation platinum compound (Figure 1A) and has shown improved stability, a broader spectrum, and higher efficacy compared with DDP and CBP (3). As a result, it has been approved by the China Food and Drug Administration (CFDA) for the treatment of inoperable, metastatic breast cancer, small cell lung cancer (SCLC), and chronic myelocytic leukemia (CML) (4). Multiple studies have demonstrated its anticancer activity against other types of cancer (5–15).

At present, very few studies have been conducted on LBP in the treatment of NSCLC. Manegold et al. (16) studied the treatment efficacy of LBP in 39 advanced NSCLC patients, and only one of the 33 evaluable cases had a partial response. This report indicated that a single treatment with LBP is hardly effective against NSCLC in Caucasian patients. Moreover, the combined treatment of LBP with vinorelbine was reported to have a response rate (RR) of 35.1% and a disease control rate (DCR) of 78.4% in an open, single-experiment group, multiple-centered phase-II clinical research trial of patients with advanced NSCLC in China (17). While the efficacy of LBP therapy in Chinese patients has been clinically validated, less has been revealed about the underlying molecular mechanism responsible for the antitumor effects of LBP in NSCLC.

The anticancer mechanism of DDP includes the induction of oxidative stress, apoptosis, enhancement of reactive oxygen species (ROS) accumulation, activation of p38 mitogen-activated protein kinases (MAPKs), and regulation of p53 (18). As a similar mechanism to that of DDP, we hypothesized that LBP might function through a similar molecular mechanism to that of DDP. P53 is a tumor suppressor gene that encodes a transcription factor that functions in response to multiple stresses, regulating the cell cycle and apoptosis by adjusting the expression profiles of many genes (19). Activated p53 can prevent the propagation of cells carrying oncogenic lesions *via* a multitude of methods, i.e., induction of growth arrest, senescence or apoptosis, modulation of tumor stroma, angiogenesis, and modification of the metabolism (20). In lung cancer, it has been revealed that p53 mutations occur in up to 46% of adenocarcinoma cases and 81% of squamous cell cancer cases (21). Platinum-based agents' effects on tumor cells with either wild-type or mutated p53 remain controversial, as some studies have reported that wild-type tumor cells were more sensitive to chemotherapeutic drugs (22), while another study has indicated that [Pt(BDIQQ)]Cl, another kind of platinum-based agent, showed similar cytotoxicity in A549 cells with or without wild-type p53 gene (23). St. Germain et al. (24) showed that the MAPK pathway was involved in the apoptosis of tumor cells induced by DDP. Additionally, DDP was reported to trigger apoptosis in colon cancer cells *via* a p53/ROS/p38MAPK/p53 loop (22). Thus, we hypothesized that the p53/ROS/p38MAPK apoptotic pathway has been involved in LBP-induced apoptosis in A549 cells with wild-type p53.

## Materials and Methods

### Compounds

LBP (Hainan Changan International Pharmaceutical Co., Ltd.) was dissolved in dimethyl sulfoxide (DMSO) to obtain 2.5 mM stock solutions (*in vitro*) or in physiological saline (*in vivo*). The stock solutions were kept frozen in aliquots at −20°C. DMSO, *N*-acetyl-L-cysteine (NAC), and SB203580 were obtained from Sigma-Aldrich (St. Louis, MO, USA). The Cell Counting Kit-8 (CCK-8) was purchased from Dojindo Molecular Technologies, Inc. (Kumamoto, Japan). The Annexin V-FITC Apoptosis Detection Kit was purchased from BD (Franklin Lakes, NJ, USA). The TUNEL staining kit was purchased from Roche (Basel, Switzerland). The ROS detection kit was purchased from Beyotime Institute of Biotechnology (Jiangsu, People's Republic of China). Antibodies against caspase-3, caspase-8, caspase-9, Bcl-2, p53, p-p38MAPK, poly-adenosine diphosphate (ADP) ribose polymerase (PARP), and GAPDH were purchased from Cell Signaling Technology (Danvers, MA, USA). Antibody against Bax was purchased from Proteintech Group Inc. (Chicago, IL, USA). GIPZ Lentiviral shRNA was purchased from Horizon Discovery (Cambridge, UK).

### Cell Lines and Cell Culture

A549, SK-MES-1, NCI-H1299, and 293T cells were acquired from Cobioer (Jiangsu, People's Republic of China). SK-MES-1 cells were cultured in MEM medium supplemented with 10% (v/v) heat-inactivated newborn calf serum (Thermo Fisher Scientific) with 1 ×10^5^ U/L penicillin G and 1 ×10^5^ U/L streptomycin in a humidified incubator at 37°C and 5% CO_2_. NCI-H1299, A549, and A549/control-shRNA cells were cultured in RPMI l640 medium supplemented with 10% (v/v) heat-inactivated newborn calf serum (Thermo Fisher Scientific) with 1 ×10^5^ U/L penicillin G and 1 ×10^5^ U/L streptomycin in a humidified incubator at 37°C and 5% CO_2_. 293T cells were cultured in DMEM medium supplemented with 10% (v/v) heat-inactivated newborn calf serum (Thermo Fisher Scientific) with 1 ×10^5^ U/L penicillin G and 1 ×10^5^ U/L streptomycin in a humidified incubator at 37°C and 5% CO_2_.

### Cell Proliferation Assay

The cytotoxic effect of LBP on NSCLC was determined by the CCK-8 assay. Cells were seeded in a 96-well culture plate at a density of 4 ×10^4^ cells/well and were cultured prior to exposure to a series of concentrations of LBP for 24, 48, and 72 h. A total of 10 μl of the CCK-8 solution was added to each well and incubated for an additional 4 h. The absorbance was measured at a 450-nm wavelength using the ELX 800 Microplate Reader (BioTek Instruments, Inc., Winooski, VT, USA). The measurement was performed three times for each concentration. The 50% inhibitory concentration (IC_50_) was determined by non-linear regression fit of the mean values of the data obtained.

### Cell Cycle Distribution Analysis

The effect of LBP on human A549 cell cycle distribution was determined by FCM analysis. Approximately 3 ×10^5^ cells were harvested at room temperature after pretreatment with various LBP concentrations for 24 h. Cells were then collected, washed twice with phosphate-buffered saline (PBS), and fixed with ice-cold 70% ethanol at 4°C overnight. After centrifugation, cells were resuspended in PBS containing 50 μg/ml RNase and incubated at 37°C for 30 min. Then, the cells were stained with 100 μl of 100 μg/ml propidium iodide (PI) and analyzed by a flow cytometer (C6; BD). The cell cycle distribution was analyzed according to the standard procedures. The distribution of cell cycle phases (G_0_/G_1_, S, or G_2_/M phase) was determined using Flow Jo.

### Apoptosis Analysis

Approximately 3 ×10^5^ A549 cells/L were harvested after pretreatment with various reagents for 24, 48, and 72 h. Then, cells were trypsinized with EDTA-free trypsinogen, washed twice in PBS, and resuspended in 400 μl of 1× binding buffer. Next, 5 μl of annexin V-FITC and 5 μl of PI were added. After incubation at room temperature for 15 min in the dark, cells were then analyzed by FCM (C6; BD).

### DAPI Staining

A549 cells were seeded in a 96-well culture plate at a density of 3 ×10^5^ cells/well, with a coverslip (containing poly-lysine) in each well. Wells were incubated overnight and assigned to groups treated with 0, 12, or 24 μM LBP. After 24 h of treatment, cells were washed with PBS and fixed with formaldehyde for 30 min. Then, cells were again washed with PBS three times for 5 min each time. To perform the staining, 2.5 μg/ml DAPI was added in the dark for 10 min. After staining, cells were again washed three times with PBS for 5 min each time. Then, 10 μl of antifade was applied on the slide before a cell-containing slip was placed on the slide. Samples were then observed with a fluorescence microscope (400×) and photographed.

### Western Blot

Western blot was performed as previously described (25). The code of caspase3 is #9662 (CST). The code of caspase9 is #9502 (CST). The code of caspase8 is #9494 (CST). The code of p-p38MAPK is #4511 (CST). The code of PARP is #9532 (CST). The code of p53 is #2527 (CST). The code of Bcl-2 is #2870 (CST). The code of GAPDH is #5174 (CST). The code of Bax is #50599-2-Ig (Proteintech). All primary antibodies and secondary were diluted to 1000 ×.

### *In vivo* Antitumor Activity

Five-weeks-old male BALB/Ca nude mice were purchased from Shanghai Laboratory Animal Center (Shanghai, China). A xenograft of A549 cells was established by inoculating viable A549 cells (10^7^ cells/100 μl PBS per mouse) into the right flanks of the nude mice. When the average tumor volume reached ~100 mm^3^, the nude mice were randomly divided into two groups (5 mice per group). The experimental group was treated with LBP (d1, d8, 12 mg/kg) *via* the tail vein, and the control group was treated with saline (d1, d8, and saline only) *via* the tail vein. The body weight of each mouse was recorded twice a week. Tumor size was measured every other day. Tumor volume was calculated with a caliper (calculated volume = shortest diameter^2^ × longest diameter/2). The mice were sacrificed after 21 days, and the tumors were excised and stored at −80°C until further analysis. The relative tumor volume (RTV) of each mouse was determined by the formula RTV = *V*_t_/*V*_0_, where *V*_t_ is the volume at each measurement and *V*_0_ is the volume at the initial treatment. The therapeutic effect of a given compound was expressed in terms of *T*/*C* (%), which was calculated by the formula *T*/*C* (%) = mean RTV of the treated group/mean RTV of the control group ×100%. All of the animal experiments were conducted following protocols approved by the animal ethics committee of the Medical School, Southeast University, and animal care was provided in accordance with institutional guidelines.

### TUNEL Analysis

Formalin-fixed tumor tissues were embedded in paraffin before being sectioned. A TUNEL system was used to evaluate apoptosis in the tumor sections that were placed on slides according to the manufacturer's protocol. Tissue sections were analyzed to detect the localized green fluorescence of the apoptotic cells and the blue fluorescence of the cell nuclei. Images were acquired and photographed using an Olympus IX51 fluorescence microscope (400×).

### Assessment of ROS

Intracellular hydrogen peroxide levels were monitored by FCM after staining with 2′,7′-dichlorodihydrofluorescein diacetate (DCFH-DA). Briefly, cells in a logarithmic growth phase (3 ×10^5^ cells in each well) were treated with various reagents for various periods of time and were then labeled with 2.5 μM DCFH-DA for 20 min. Next, the cells were trypsinized, washed with PBS, and then analyzed by FCM (Becton Dickinson). The percentage of cells displaying increased dye uptake was used to reflect an increase in ROS level.

### Construction of the P53 shRNA Expression Vector and Establishment of Stable Clones Expressing p53 shRNA

Specific shRNA and control shRNA were designed and synthesized by Nanjing Kebai Biotech. Co. Ltd. (Nanjing, China). A BLAST search was performed with the National Center for Biotechnology Information (NCBI) database to ensure that the shRNA constructs were targeting only human p53. The sequence of the p53 shRNA was 5′-TACACATGTAGTTGTAGTG-3′, while the sequence of the nonsense-p53 shRNA (NS-p53-shRNA) was 5′-TTCTCCGAACGTGTCACGT-3′. The oligonucleotides were annealed and cloned into the pGIPZ-CMV-GFP-Puro, as described by the manufacturer. A total of 2.5 μg of pGIPZ constructs containing shRNAs and 7.5 μg of packaging plasmid were used to transfect 293T cells following the calcium phosphate precipitation method. After 48 h, lentiviruses containing targeted gene shRNA were collected and used to transfect A549 cells according to the manufacturer's instructions.

### Statistical Analysis

Statistical analysis was performed using the SPSS 22.0 software (IBM Corporation, Armonk, NY) package for Windows. Statistical significance was calculated using unpaired Student's *t*-test, with *P* < 0.05 indicating statistical significance.

## Results

### LBP Inhibits the Proliferation of A549, SK-MES-1, and NCI-H1299 Cells and Induces G1 Cell Cycle Arrest in A549 Cells

The CCK-8 assay was performed to evaluate the effects of LBP on the proliferation of A549, NCI-H1299, and SK-MES-1 cells. The cells were treated with LBP at concentrations of 270, 90, 30, 10, 3.3, 1.1, 0.37, or 0.123 μM for 24, 48, or 72 h. As shown in Figure 1B, LBP reduced the proliferation of cells in a dose- and time-dependent manner. The IC_50_ values of LBP for SK-MES-1 cells were 63.47 ± 1.03, 15.77 ± 1.09, and 12.0 ± 1.08 μM when the cells were treated for 24, 48, and 72 h, respectively. The IC_50_ values for NCI-H1299 cells were 67.26 ± 1.06, 17.98 ± 1.05, and 7.85 ± 1.01 μM for the 24-, 48-, and 72-h treatment times, respectively, while the IC_50_ values for A549 cells were 11.69 ± 1.05, 5.02 ± 1.11, and 2.43 ± 1.01 μM for the 24-, 48-, and 72-h treatment times, respectively. It seems that LBP had a notably low IC_50_ in A549 cells with wild-type p53. Therefore, the A549 cell line was chosen for the next experiments to investigate the anticancer mechanism of LBP. Based on the IC_50_ of LBP, 6 and 12 μM were selected to be the experimental concentrations for the cell cycle arrest experiments, while 12 and 24 μM concentrations were selected for apoptosis detection, DAPI stain, and Western blot of A549 cells.

FCM analysis was performed to assess the cell cycle of cells treated with low concentrations of LBP (6 and 12 μM). After 24 h of treatment, 72.03 ± 1.53% of the cells remained in G1 phase under 6 μM LBP treatment (Figure 1C-b), while 73.78 ± 1.26% of the cells remained in G1 phase under 12 μM LBP treatment (Figure 1C-c). In the control group (Figure 1C-a), only 47.47 ± 1.59% of cells were detected in this phase. Additionally, the percentage of cells in the G2 or S phases was strikingly decreased in the experimental groups (*P* < 0.05). Our data showed that LBP could induce G1 phase cell cycle arrest at both 6 and 12 μM concentrations.

### LBP Induces Apoptosis in A549 Cells Both *in vitro* and *in vivo*

**Figure 2:**
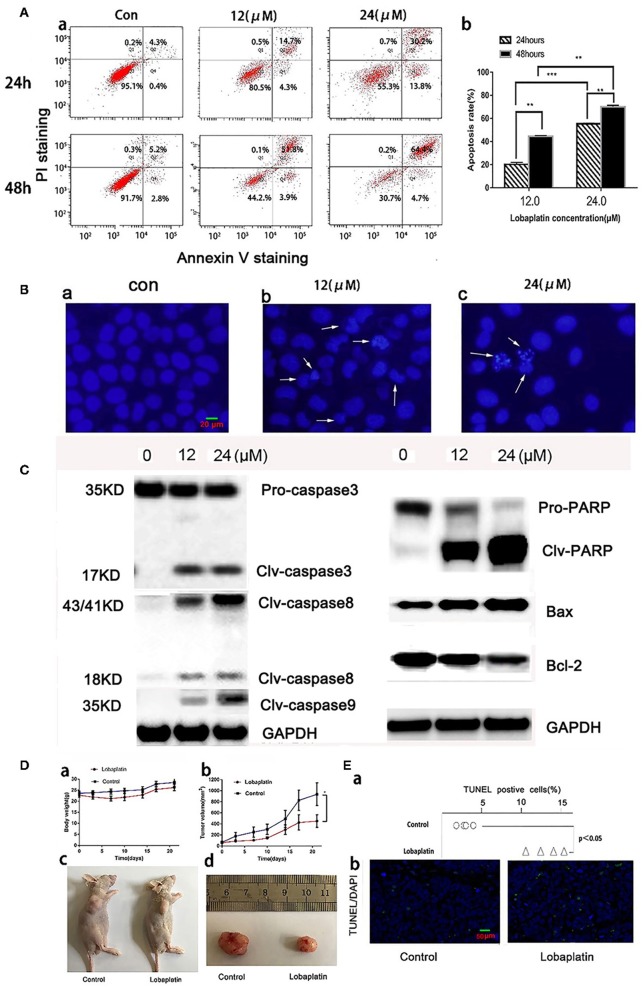
Induction of apoptosis in A549 cells by LBP *in vitro* and *in vivo*. **(A,a)** A549 cells were treated with LBP at the indicated concentrations for 24 and 48 h. The apoptosis rates were calculated by adding the percentages of Q2 and Q4, which appeared to be annexin V-positive. **(b)** The apoptosis rate was analyzed statistically. The data are expressed as means ± SD. **P < 0.01 and ***P < 0.001. **(B)** After being treated with LBP at concentrations of 0 μM **(a)**, 12 μM **(b)**, and 24 μM **(c)**, A549 cells were stained with DAPI at a concentration of 2.5 μg/ml for 10 min and were then observed through a fluorescence microscope (400×) for morphological changes in their nuclei. **(C)** Expression levels of PARP, Bax, Bcl-2, and clv-caspase-3/8/9 affected by LBP at concentrations of 0, 12, and 24 μM for 24 h. GAPDH was used as an internal reference. **(D)** The antitumor effect of LBP on A-549 human xenograft models. **(a)** The average body weight of each group was expressed as the mean ± SD. **(b)** Tumor size comparison between the treatment group (*n* = 5), which was given LBP at 12 mg/kg *via* caudal vein injection, and the control group, which was treated with normal saline (*P* < 0.05). **(c)** Image of mice with A-549 xenograft tumors after 21 days of treatment. **(d)** At the experimental end point, tumors were dissected and photographed. For **(c,d)**, only one mouse is shown as an example for each group. **(E,a)** The TUNEL-positive rate of each tumor was analyzed. **(b)** Representative images of TUNEL/DAPI staining of tumor tissues of the different groups (magnification 200×). *P < 0.05.

The Annexin V-FITC/PI Apoptosis Detection Kit was used to quantitatively determine whether LBP could induce A-549 cell apoptosis. The result showed that after treatment with 12 μM LBP for 24 h, 20.2 ± 1.3% of the experimental A549 cells developed apoptosis, while 44.5 ± 1.6% of the cells were affected at a concentration of 24 μM LBP. After we doubled the treatment time, apoptotic proportion of A549 cells treated with LBP at concentrations of 12 and 24 μM increased to 55.3 ± 1.2% and 70.0 ± 0.9%, respectively, suggesting that LBP could induce A549 cell apoptosis in a time- and dose-dependent manner (*P* < 0.05, Figure 2A). Next, we performed DAPI staining to observe the morphological changes of the cell nuclei during the process of LBP-induced apoptosis. After being treated with 12 μM LBP for 24 h, nuclei were observed to be condensed and deformed. When cells were treated with 24 μM LBP for 24 h, fewer nuclei were found intact in the same field of vision than were found in the cells treated with 12 μM LBP, and apoptosis bodies were found (Figure 2B). We then performed Western blot to study the expression levels of apoptosis-related proteins. After being treated with LBP at 12 or 24 μM LBP for 24 h, expression levels of clv-PARP, Bax, and clv-caspase-3,8,9 were upregulated and Bcl-2 was downregulated all in a dose-dependent manner (*P* < 0.05, Figure 2C).

To study LBP's antitumor efficacy against A549 cells *in vivo*, we further generated a model of nude mice bearing human A549 tumors. The data showed that the experimental group weighed slightly less than the control group did, but there was no significant difference (*P* > 0.05). The mice lost some weight right after each time they were treated with LBP, which indicated that LBP might cause a mild gastrointestinal reaction (Figure 2D-a). By the end of the experiment, the average of the LBP group tumor was 451 ± 113 mm^3^ while it was 936 ± 205 mm^3^ in the control group (Figure 2D-b). The *T*/*C* value was 55.2%, and the tumor inhibition rate was 47.9% and Supplementary Figures 1, 2 (Figures 2D-c,d), suggesting that LBP could inhibit the growth of the A549 tumor. TUNEL/DAPI staining indicated the apoptosis rate of 15.5 ± 2.1% in the treatment group, whereas the percentage was only 4.8 ± 0.5% in the control group (*P* < 0.05) (Figures 2E-a,b). Taken together, our results indicated that LBP inhibited the growth of A549 tumors in mice by inducing the apoptosis.

### LBP Induces ROS in A549 Cells

**Figure 3:**
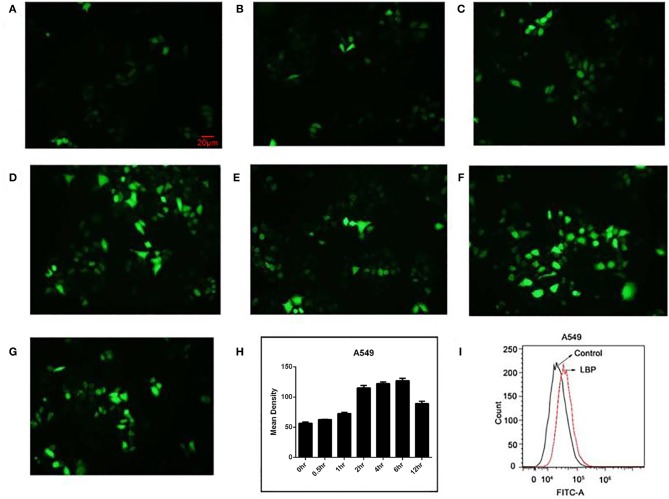
ROS release in A549 cells after LBP treatment. With DCFH-DA staining, the levels of ROS are shown at **(A)** 0 h, **(B)** 0.5 h, **(C)** 1 h, **(D)** 2 h, **(E)** 4 h, **(F)** 6 h, and **(G)** 12 h. **(H)** The levels of ROS were analyzed statistically. **(I)** A549 cells were treated with 24 μM LBP for 6 h. FCM analysis of intracellular ROS using a DFCH-DA probe.

To test whether ROS are involved in the pathway through which LBP induces A549 cell apoptosis, we examined ROS levels in A549 cells after treating the cells with 24 μM LBP. We performed the ROS analysis with both DCFH-DA staining analysis with a fluorescence microscope and FCM. From the resulting images, it can be inferred that ROS release increased as the treatment time increased (Figures 3A–G), with an apparent burst at 6 h (*P* < 0.05) (Figure 3H). FCM analysis also confirmed this finding (*P* < 0.05) (Figure 3I).

### P53 and p38 MAPK Are Upregulated During LBP-Induced Apoptosis

**Figure 4:**
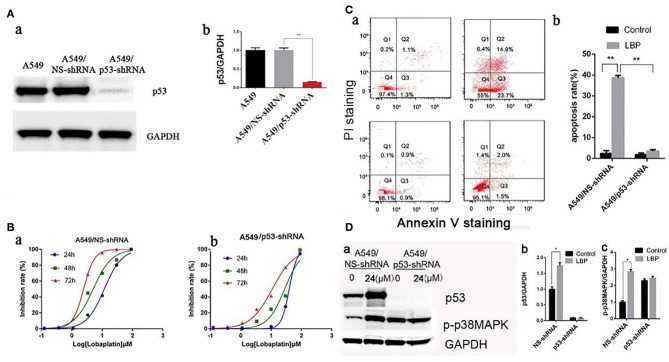
LBP induces apoptosis by upregulating p53 and p38MAPK. **(A)** Establishment of stable p53-knockdown clones. The plasmid containing the p53-shRNA sequence was constructed and transfected into A549 cells, and the stable lines harboring vector control (A549/NS-shRNA) or p53-shRNA vector were isolated as described in the Materials and Methods section. The p53 protein expression levels were determined by Western blot. **(B)** Cells were exposed to various concentrations (0.00123–90 μM) of LBP for 24, 48, or 72 h followed by analysis with a CCK-8 assay. **(a)** A549/NS-shRNA, **(b)** A549/p53-shRNA. **(C,a)** A549/NS-shRNA and A549/p53-shRNA cells were treated with LBP at the indicated concentrations for 24 h. The apoptosis rates were calculated by adding the percentages of Q2 and Q4, which appeared to be annexin V-positive. **(b)** The apoptosis rate was analyzed statistically. The data are expressed as the means ± SD. **(D,a)** Western blot results detecting expression levels of p53 and p-p38MAPK in A549/NS-shRNA and A549/p53-shRNA cells treated with 24 μM LBP for 24 h. GAPDH was used as an internal reference. **(b)** Protein expression levels (relative to GAPDH) were determined. The data are expressed as the means ± SD. *P < 0.05 and **P < 0.01.

To determine whether LBP-induced apoptosis requires p53, stable cell lines transfected with the plasmid-based shRNA expression system targeting p53 mRNA were established, and the sensitivity of these cell lines to LBP was then surveyed. As shown in Figure 4A, the p53 protein level of A549/p53-shRNA was efficiently knocked down compared to the expression in the A549 parental control or A549/NS-shRNA (vector control) cells. Subsequently, A549/NS-shRNA and A549/p53-shRNA cells were exposed to LBP at concentrations ranging from 0.0123 to 90 μM for 24, 48, or 72 h (Figure 4B). The IC_50_ of LBP for A549/p53-shRNA cells was 39.54 ± 1.02 μM for the 24-h treatment, 27.89 ± 1.13 μM for the 48-h treatment, and 10.93 ± 1.08 μM for the 72-h treatment. However, the IC_50_ of LBP for A549/NS-shRNA cells was 11.48 ± 1.12 μM for the 24-h treatment, 5.14 ± 0.94 μM for the 48-h treatment, and 2.49 ± 0.90 μM for the 72-h treatment. Compared to the A549 cells, the A549/NS-shRNA cells showed similar sensitivity to LBP (*P* > 0.05). Hence, A549/p53-shRNA cells with p53 knockdown became less sensitive to LBP, as shown by their higher IC_50_ values (*P* < 0.05). Sequentially, FCM analysis in association with annexin V-PI double staining was performed to investigate the apoptosis of A549/NS-shRNA and A549/p53-shRNA cells treated with LBP. The results showed that after 24 h, 38.6 ± 1.1% of the 24 μM-LBP-treated A549/NS-shRNA24h cells underwent apoptosis, but no significant apoptosis observed in A549/p53-shRNA cells (Figure 4C). Our findings suggested that A549/p53-shRNA cells were more resistant to LBP-induced apoptosis than A549/NS-shRNA cells (*P* < 0.05). To further evaluate whether LBP could also modify the expression level of p38MAPK, expression levels of p53 and p-p38MAPK were measured in A549/NS-shRNA and A549/p53-shRNA cells that were treated with 24 μM LBP for 24 h. As shown in Figure 4D, LBP could upregulate the expression levels of p53 and p-p38MAPK in A549/NS-shRNA cells (*P* < 0.05) but not in A549/p53-shRNA cells (*P* > 0.05). Interestingly, p-p38MAPK was found to have a higher expression level in A549/p53-shRNA cells than in A549/NS-shRNA cells with no LBP treatment.

### LBP Induces ROS Generation in p53-Dependent Manner in A549 Cells

**Figure 5:**
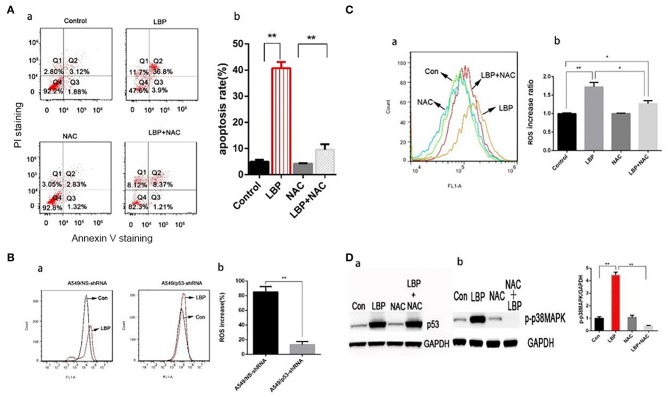
LBP-induced p53 and p-p38MAPK expression, ROS release, and apoptosis are affected by NAC. **(A,a)** A549 cells were treated with 5 mM NAC for 4 h and 5 mM NAC for 4 h before treatment with 24 μM LBP for 24 h, followed by FCM analysis. The apoptosis rates were calculated by adding the percentages of Q2 and Q4. **(b)** The apoptosis rate was analyzed statistically. The data are expressed as the means ± SD. *P < 0.05 and **P < 0.01. **(B)** FCM analysis of intracellular ROS using the DFCH-DA probe. A549/NS-shRNA and A549/p53-shRNA cells were treated with LBP at a concentration of 24 μM for 6 h. The value shown in **(b)** is the percentage of increase. **(C)** FCM analysis of intracellular ROS using the DFCH-DA probe. A549 cells were treated with 5 mM NAC for 4 h and 5 mM NAC for 4 h before treatment with 24 μM LBP for 6 h. Con indicates the control group. *P < 0.05, **P < 0.01. **(D)** Western blot detecting **(a)** p53 and **(b)** p-p38MAPK expression levels with and without NAC. A549 cells were pre-treated with 5 mM NAC for 4 h before treatment for 24 h with 24 μM LBP.

Based on previous results, we applied an ROS inhibiting agent, NAC, to determine whether ROS are crucial for LBP-induced apoptosis. After pretreatment with NAC, cells were incubated with 24 μM LBP for 24 h, followed by FCM. As shown in Figure 5A, the apoptosis rate was reduced from 40.7 ± 2.4% to 9.6 ± 2.1% (*P* < 0.05). Our findings suggested that ROS participated in the process of LBP-induced apoptosis.

With DCFH-DA staining followed by FCM, we measured the ROS levels in A549/NS-shRNA and A549/p53-shRNA cells treated with LBP at a concentration of 24 μM for 6 h. While only a slight upregulation of ROS release occurred in the A549/p53-shRNA cells, A549/NS-shRNA cells generated a much higher level of ROS under LBP treatment. The percentage of ROS released was significantly higher in the A549/NS-shRNA cells than in the A549/p53-shRNA cells (*P* < 0.05, Figure 5B). NAC can partially suppress LBP-induced ROS release (*P* < 0.05, Figure 5C). To investigate the precise connection between p53, ROS, and p38MAPK in the pathway of LBP-induced apoptosis, we applied NAC and SB203580, a p38MAPK inhibiting agent, to the cells before LBP treatment to monitor the expression levels of p53 and p38MAPK. The results were shown in Figure 5D. It is implied that LBP at a concentration of 24 μM could upregulate the expression of p53 and p-p38MAPK (*P* < 0.05). Treatment with NAC prevented p-p38MAPK activation (*P* < 0.05), while p53 levels remained unchanged (*P* > 0.05). This result suggested that the p53/ROS/p38MAPK pathway was involved in the LBP-induced apoptosis.

### Activation of p38MAPK by LBP Is Mediated by p53-Induced ROS

**Figure 6:**
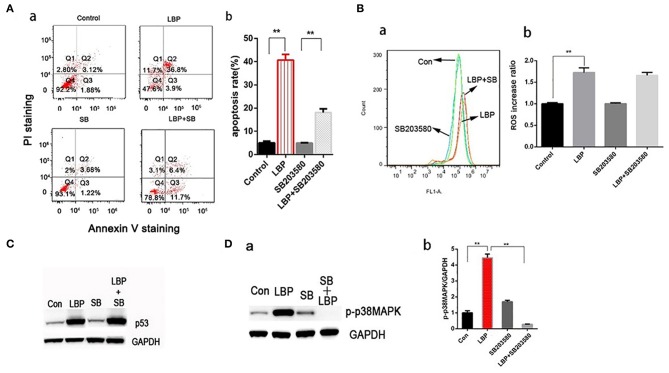
P-p38MAPK is involved in the mechanism of LBP-induced apoptosis. **(A,a)** A549 cells were treated with 12.5 μM SB203580 for 1 h and 12.5 μM SB203580 for 1 h before treatment with 24 μM LBP for 24 h followed by FCM. **(b)** The apoptosis rate was analyzed statistically. The data are expressed as the means ± SD. *P < 0.05 and **P < 0.01. **(B)** FCM analysis of intracellular ROS using the DFCH-DA probe. A549 cells were treated with 12.5 μM SB203580 for 1 h and 12.5 μM SB203580 for 1 h before treatment with 24 μM LBP for 6 h. Con indicates the control group. *P < 0.05, **P < 0.01. **(C)** Western blot detecting p53 expression levels with and without SB203580 treatment. A549 cells were pre-treated with 12.5 μM SB203580 for 1 h before treatment for 24 h with 24 μM LBP. **(D)** Western blot detecting p-p38MAPK expression levels with and without SB203580 treatment. A549 cells were pretreated with 12.5 μM SB203580 for 1 h before treatment for 24 h with 24 μM LBP.

P38MAPK plays vital roles in many stress reaction pathways in human cells. P38MAPK has been reported to be involved in DDP's antitumor mechanism (22). In our experiment, LBP at a concentration of 24 μM can induce a significant upregulation of p-p38MAPK in A549 cells (*P* < 0.05), which was inhibited by the application of SB203580 (*P* > 0.05, Figure 6D). To further examine the role of p38MAPK in LBP-induced A549 cell apoptosis, we applied SB203580 to the cells before they were treated with 24 μM LBP for 24 h followed by FCM for apoptosis analysis. The results showed that pretreatment with SB203580 reduced the LBP-induced apoptosis rate in A549 cells from 40.7 ± 2.4% to 18.1 ± 1.6% (*P* < 0.05, Figure 6A). Our findings indicated that p38MAPK participates in the process of LBP-induced apoptosis. Next, we performed DCFH-DA staining followed by FCM to detect ROS accumulation in A549 cells treated with or without SB203580 before LBP treatment. The results showed that SB203580 failed to alleviate the accumulation of ROS induced by LBP (*P* > 0.05, Figure 6B). Moreover, our findings indicated that SB203580 could not inhibit the upregulation of p53 triggered by 24 μM LBP (*P* > 0.05, Figure 6C).

## Discussion

In this study, we observed that LBP can induce the apoptosis of NSCLC cells with different p53 genotypes, and A549 cells with wild-type p53 were the most sensitive to LBP-induced apoptosis. LBP was also found to reduce the tumor burden in the A549 xenograft model. Importantly, the underlying mechanisms of LBP-induced apoptosis were revealed, involving the p53/ROS/p38MAPK signaling pathway.

Lung cancer cells with different p53 genotypes were treated with LBP. A549 cells with wild-type p53 were more sensitive to LBP. Additionally, we measured the 48-h IC_50_ values of LBP for NCI-H1299 and A549 cells, which were 18.00 ± 1.05 and 5.02 ± 1.11 μM, respectively. In comparison, DDP was reported to have IC_50_ values of 25.21 ± 4.38 and 21.88 ± 3.21 μM for NCI-H1299 and A549 cells, respectively (26). Thus, LBP showed more powerful effects than DDP on A549 and NCI-H1299 cells.

Cell cycle arrest is one of the mechanisms for cell growth inhibition induced by many anticancer drugs. Many platinum-based drugs, including DDP, CBP, oxaliplatin, and nedaplatin (27–29), have been found to induce cell cycle arrest. In this study, LBP was also found to trigger G1 phase cell cycle arrest. Wang et al. (14) found that LBP could induce G1 phase cell cycle arrest in cholangiocarcinoma cells. In SMMC-7721 cells, LBP was found to cause arrest in the G1 phase within 24–48 h of treatment and G2/M arrest within 36–48 h of treatment (30). It was also reported that 3 μM LBP led to S phase arrest in A549 cells (31). In general, LBP can induce cell cycle arrest, which may vary by phases according to different cell types, dosages, and/or treatment times.

Apoptosis is one of the most important anticancer mechanisms (32). Many chemotherapeutic compounds, like DDP (26) and CBP (33), have been found to inhibit tumor growth by inducing apoptosis. With DAPI staining, we observed condensation and margination of chromatin in the A549 cells that were treated with LBP for 24 h. In the FCM analysis, the LBP-induced apoptosis rates were found to be dose- and time-dependent. TUNEL staining confirmed that LBP could induce A549 cell apoptosis *in vivo*. LBP can also induce a dose-dependent upregulation of PARP, which was also proven to be regulated by DDP (26). The expression levels of clv-caspase-3/8/9 were also indicated by the Western blot results to be elevated by LBP in A549 cells in a dose-dependent manner. Thus, both internal and external apoptotic pathways are employed by LBP to induce A549 cell apoptosis.

In the *in vivo* study, LBP could effectively inhibit tumor growth in mice bearing A549 tumors through the induction of apoptosis, with a relative *T*/*C* of 55.2% and an inhibitory rate of 47.9%. Harstrick et al. (34) applied LBP to mice bearing H12.1 tumors. LBP was applied at a dosage of 12 mg/kg when the tumor size reached 1–1.5 cm^3^ on days 1, 3, and 5, yielding a *T*/*C* value of 39.1%. It seems that the effectiveness of LBP depends on the timing, frequency, and dosage of treatment; these factors will require further investigation.

In normal physiological conditions, ROS are natural by-products of aerobic respiration and metabolism. Some anticancer agents, such as gemcitabine (35), pemetrexed (36), gefitinib (37), paclitaxel (38, 39), vinorelbine (40), docetaxel (41), CBP (42), and oxaliplatin (43), work by generating ROS. The overaccumulation of ROS induces cell apoptosis through internal and external apoptotic pathways (44). LBP can upregulate the ROS level in human BGC-823 stomach cancer cells (6). Our study showed that LBP at a concentration of 24 μM was able to trigger ROS accumulation in A549 cells, with a burst at 6 h. The LBP-induced ROS release and apoptosis in A549 cells could be partially attenuated by NAC. This finding indicates that ROS play a role in the A549 cell apoptosis induced by LBP.

P53 is thought to be involved in the maintenance of genome integrity. DNA damage can trigger a rapid upregulation of p53 expression (45). Once activated, p53 is able to affect a number of transcription factors, such as ROS (19) and MAPKs (18). MAPKs, including ERK, p38, and JNK, are components of signaling cascades that respond to extracellular stimuli by targeting transcription factors, resulting in the regulation of gene expression. DDP has also been reported to employ both p38MAPK and JNK pathways to kill human cervical cancer cells (46). CBP can induce the apoptosis of human cervical cancer cells through the p53/ERK pathway (47). LBP has an anti-tumor mechanism where it binds to guanine and adenine in double-strand DNA, creating a Pt–DNA conjugate compromising genome integrity, which is similar to that of DDP (48). Thus, we continued to examine whether p53 and p38MAPK are involved in the induction of apoptosis by LBP. First, our experiments showed that treatment with 24 μM LBP for 24 h elevated p53 expression in A549 cells. LBP had a much higher IC_50_ for A549/p53-shRNA cells than for A549/NS-shRNA cells. Moreover, LBP at a concentration of 24 μM resulted in an apoptosis rate of 45% in p53/NS-shRNA cells, while no apparent apoptosis was observed in A549/p53-shRNA cells. Our findings demonstrated that A549 cells were less sensitive to LBP without the presence of the p53 gene. In comparison, Suntharalingam et al. (23) reported that the IC_50_ of a novel platinum-based agent, [Pt(BDIQQ)]Cl, was the same in A549 and A549/p53^−/−^ cells, which indicates that the inhibition of tumors is p53-independent. Gefitinib, reported by Chang et al. (49), was more active in A549 cells with wild-type p53 than on A549/p53-shRNA cells, while NCI-H1299 with p53^+/+^ cells was sensitive to gefitinib. In another study (30), Huh-7 cells with a mutated p53 gene were less sensitive to LBP than were SMMC-7721 and Bel-7402 cells but showed similar sensitivity to HePG2 cells; all three of these cell lines are liver cancer cells with wild-type p53. Based on these studies, it can be inferred that whether p53 is key to cancer cells' sensitivity to platinum-based drugs relies on the types of cells and/or drugs. Overall, p53 is required for LBP-induced A549 apoptosis. Second, we found that LBP could not lead to an upregulation of p-p38MAPK in A549/NS-shRNA cells. However, in A549/p53-shRNA cells, the p-p38MAPK expression level was noticeably higher than in A549/NS-shRNA cells, an observation that requires further investigation. We found that NAC and SB203580 were able to downregulate the p-p38MAPK expression level in A549/p53-shRNA cells. SB203580 showed no interference with the upregulation of p53 and ROS induced by LBP. However, pretreatment with SB203580 reduced the LBP-induced apoptosis rate in A549 cells. Overall, it is indicated that p38MAPK is also required for the LBP-induced apoptosis of A549 cells. At last, we found that a slight upregulation of ROS release occurred in the A549/p53-shRNA cells after LBP treatment. The expression of p53 is not affected by the application of NAC. LBP can also trigger the upregulation of p38MAPK, which is inhibited by applying NAC to the cells. By far, we draw the conclusion that LBP induced A549 apoptosis through the p53/ROS/p38MAPK pathway. This pathway has been proved to be involved in the induction of HCT-116 cell apoptosis by DDP (22) and in the inhibition of breast cancer metastasis by theaflavin (50). Both studies reported a downregulation of p53 after the treatment with SB203580. But in this study, no such effect of SB203580 on p53 expression was found, which is consistent with results by Sanchez-Prieto et al. (51). The difference still needs to be well-investigated.

In summary, our findings revealed that LBP could arrest the cell cycle in the G1 phase in A549 cells, significantly inhibit the growth of A549 cells *in vitro* and *in vivo*, and induce cell apoptosis by upregulating the expression levels of Bax, PARP, clv-caspase-3,−8, and−9 and downregulating the expression of Bcl-2. We verified for the first time that the p53/ROS/p38MAPK signaling pathway was involved in LBP-induced apoptosis. Taken together, our findings clearly concluded that LBP is a promising candidate for the treatment of NSCLC.

## Data Availability

The raw data supporting the conclusions of this manuscript will be made available by the authors, without undue reservation, to any qualified researcher.

## Ethics Statement

All of the animal experiments were conducted following protocols approved by the Animal Ethics Committee of the Medical School, Southeast University, and animal care was provided in accordance with Institutional Guidelines.

## Author Contributions

HoZ, RC, and XZ designed the study. HoZ and XW performed the experiments. HoZ, HaZ, and JC analyzed the data. HoZ and RC wrote the manuscript.

### Conflict of Interest Statement

The authors declare that the research was conducted in the absence of any commercial or financial relationships that could be construed as a potential conflict of interest.

## Supplementary Material

Supplementary Figure 1 Image of mice of LBP treatment group with A549 xenograft.

Supplementary Figure 2 Image of mice of control group with A549 xenograft.
