# Supplementary figures and images for: Lobaplatin-Induced Apoptosis Requires p53-Mediated p38MAPK Activation Through ROS Generation in Non-Small-Cell Lung Cancer
Source: Front Oncol. 2019 Jul 24;9:538. doi: 10.3389/fonc.2019.00538 (PMC6689983; doi:10.3389/fonc.2019.00538)

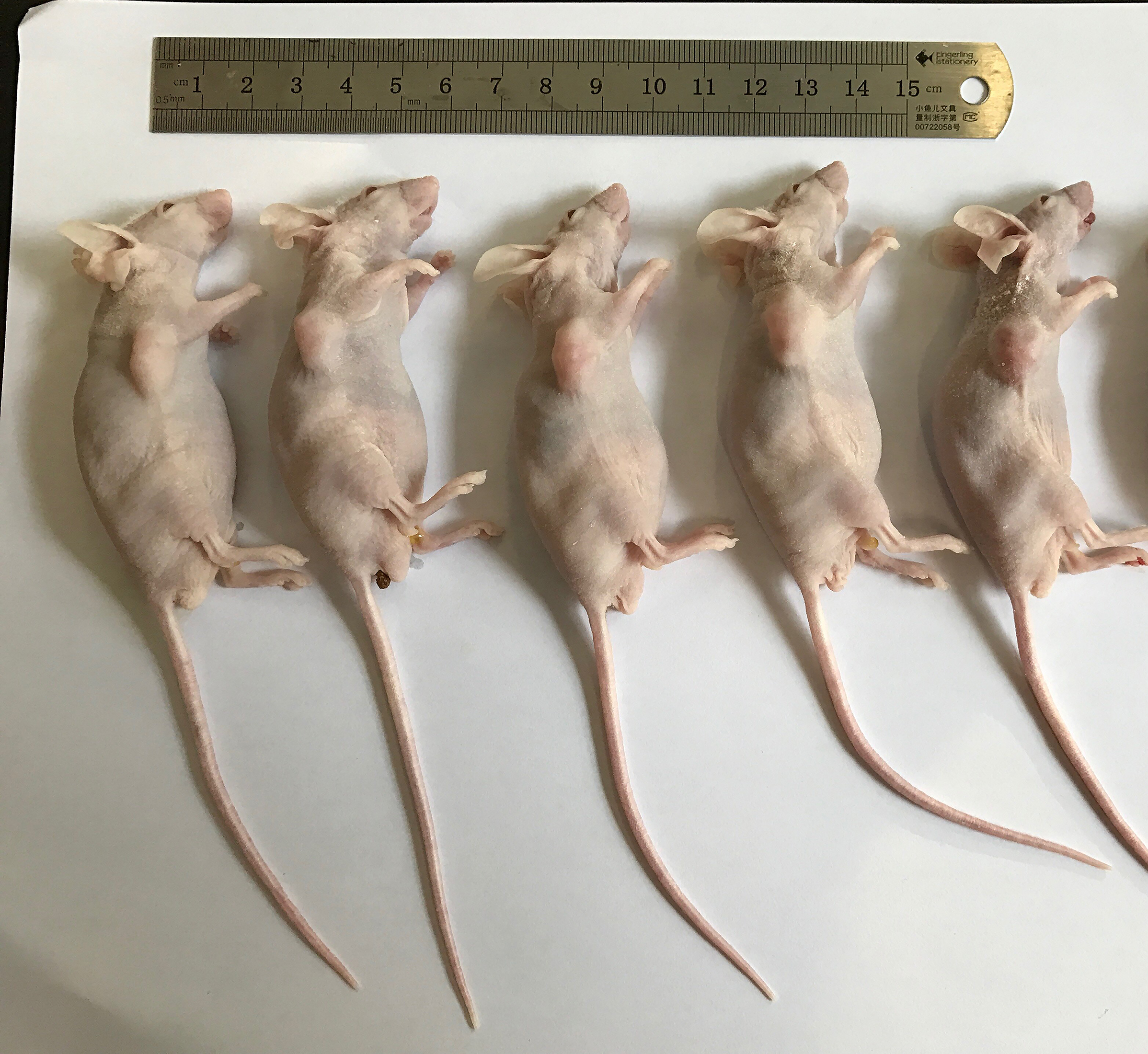

Supplement: Supplementary Figure 1 — Image of mice of LBP treatment group with A549 xenograft. [file Image_1.jpeg]

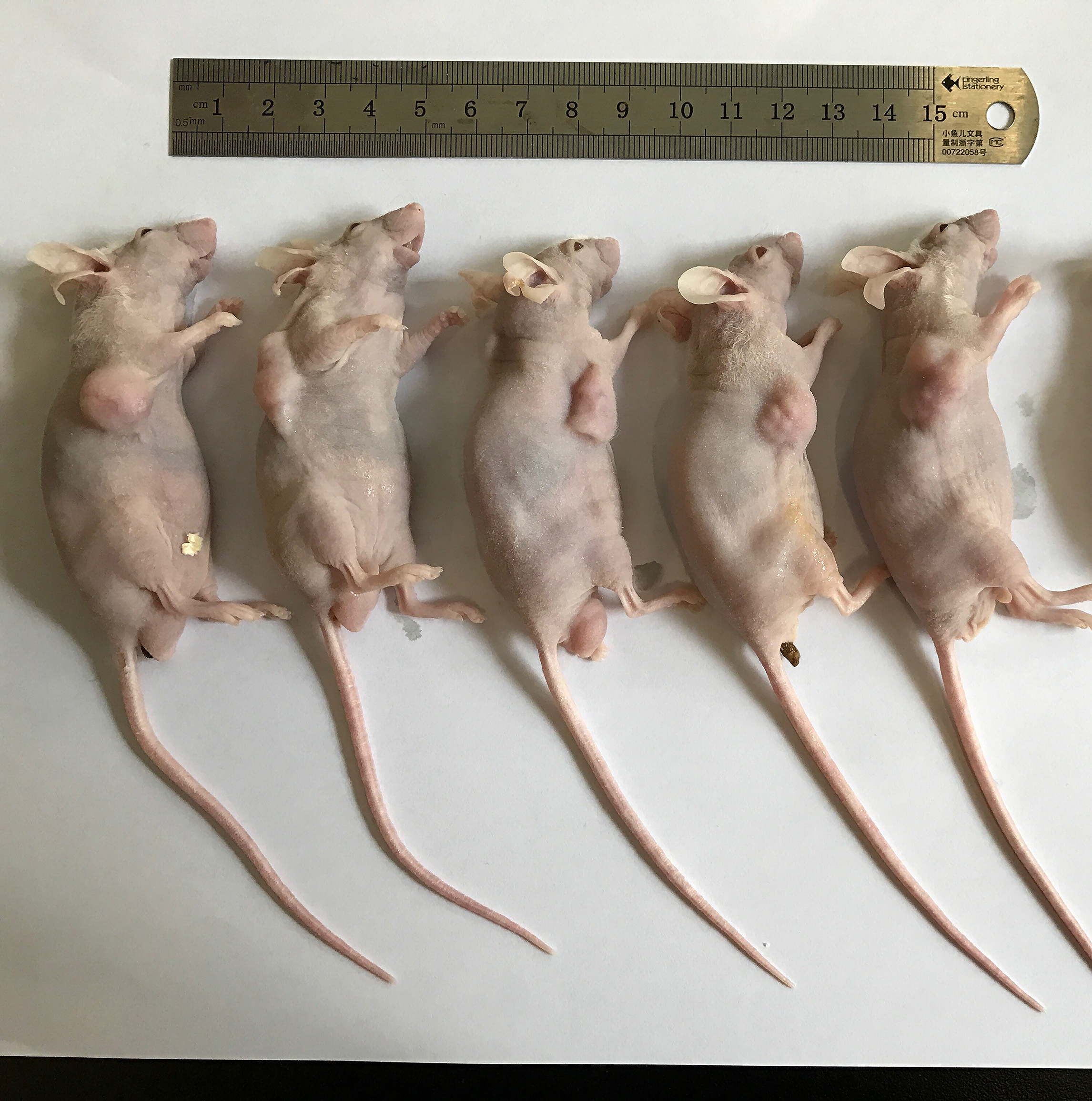

Supplement: Supplementary Figure 2 — Image of mice of control group with A549 xenograft. [file Image_2.jpeg]
